# The Ethical Dilemma of Safety Versus Self-Determination in Long-Term Care Community Residents During COVID-19

**DOI:** 10.1093/geroni/igab046.607

**Published:** 2021-12-17

**Authors:** Rebecca Davis, Cheryl Monturo, Maria O'Reilly, Diana Sturdevant

**Affiliations:** 1 Grand Valley State University, Grand Rapids, Michigan, United States; 2 West Chester University, West Chester, Pennsylvania, United States; 3 Central Queensland University, Bundaberg, Queensland, Australia; 4 University of Oklahoma HSC, Oklahoma City, Oklahoma, United States

## Abstract

The pandemic profoundly affected the care of older adults in long term care communities (LTCC) across the world. More than one third of pandemic deaths were linked to nursing homes. Most nations and states had strict guidelines on visitation, with many, especially in the United States, totally prohibiting visitation for over an entire year. Well-intentioned measures to protect through isolation caused a profound ethical tension between safety and self-determination. The aim of the project was to examine this dilemma using a case study and the Madison Collaborative Ethical Reasoning in Action Framework. Eight key questions of fairness, outcomes, rights, responsibilities, character, liberty, empathy, and authority were applied in the context of federal and state mandates in the US and Australia. Results highlighted issues of ageism, paternalism vs empathy, regulatory vs family authority, a focus on short-term outcomes while forfeiting long-term outcomes, community responsibilities to the resident trumped individual resident rights, the potential loss of community character in lieu of basic care provision, a loss of personal freedoms, and the emphasis of physical well-being over holistic well-being. The results of this analysis can inform future policy and provide lessons learned for the future.

